# Small Bowel Obstruction: A Rare Presentation of the Inferior Pancreaticoduodenal Artery Pseudoaneurysm Bleed

**DOI:** 10.7759/cureus.16943

**Published:** 2021-08-06

**Authors:** Hasan M Zerti, Muhammad Sabih Saleem, Yakub I Khan, Zaid Iqbal, Hammed Ninalowo

**Affiliations:** 1 Internal Medicine, Geisinger Wyoming Valley Medical Center, Wilkes Barre, USA; 2 Internal Medicine, Geisinger Wyoming Valley, Wilkes Barre, USA; 3 Gastroenterology, Geisin, Wilkes Barre, USA; 4 Internal Medicine, Wright Cen, Scranton, USA; 5 Intervention Radiology, Geisin, Wilkes Barre, USA

**Keywords:** pseudoaneurysm, gastroduodenal artery, inferior pancreaticoduodenal artery, intra-abominal bleed, intramural hematoma

## Abstract

Intra-abdominal and intramural hematomas are well-known complications of pseudoaneurysms. We present a case of small bowel obstruction as a result of external mechanical compression from hematoma. Bleeding was localized to the pseudoaneurysm of the gastroduodenal artery and inferior pancreaticoduodenal artery. Angiography was used to control the bleeding with coil embolization. This rare clinical manifestation represents just one of the symptoms associated with pseudoaneurysms of the gastrointestinal tract. Therapeutic options are discussed along with a review of the literature.

## Introduction

Visceral arterial aneurysms of the splenic, renal, hepatic, or mesenteric arteries are associated with a wide variety of clinical presentations. A pseudoaneurysm represents arterial dilatation with disruption of the vessel wall due to injury from trauma, inflammation, infection, or connective tissue disease, whereas true aneurysms are the result of vessel wall malformations [1]. Perivascular tissue, inflammatory fibrosis, and thrombi often constitute the wall of a pseudoaneurysm [2]. We report a case where an intramural hematoma of the duodenum as a result of a pseudoaneurysm from the gastroduodenal artery (GDA) from the celiac trunk and inferior pancreaticoduodenal artery (IPDA), resulted in symptoms of small bowel obstruction by compressing the second and third portions of the duodenum. A review of the literature recounts cases where intramural hematomas cause compression of the bowel; however, this case is unique as it represents an intramural hematoma not precipitated by trauma or identified infection.

## Case presentation

A 62-year-old woman with a past medical history of degenerative joint disease, cholecystectomy, total abdominal hysterectomy, and multiple back surgeries, presented to a peripheral hospital with a sudden onset of acute epigastric abdominal pain associated with nausea and vomiting. Further investigation revealed intra-visceral hemorrhage from the GDA. She was transferred to our hospital where the patient underwent elective airway protective intubation and further diagnostic CT-guided angiography.

The patient underwent embolization of the GDA and IPDA for bleeding pseudoaneurysms. Interventional radiology findings included pseudoaneurysms from the GDA and IPDA axis. The patient was also noted to have celiac artery stenosis and a 1.6-cm splenic artery aneurysm.

 The patient required a second angiography by Interventional radiology for an acute drop in hemoglobin and hypotension. Further embolization of the residual vessels in the peri duodenal area was performed. The patient was subsequently extubated, was able to tolerate diet, and was discharged home.

Three weeks later the patient presented to the emergency room with abdominal pain, nausea, and vomiting. CT of the abdomen-pelvis revealed dilatation of the second and third portions of the duodenum. After an extensive discussion among the gastroenterology, surgical, and interventional radiology services, nasogastric tube decompression with bowel rest and medical management was pursued. An abdominal x-ray was performed to demonstrate the extent of the stenosis, which revealed narrowing of the second and third portions of the duodenum, secondary to known extrinsic compression from a hematoma, with free flow of contrast through the segmental narrowing into the distal duodenum.

As the patient’s nausea and abdominal pain improved, the nasogastric tube was taken out and the patient underwent an esophagogastroduodenoscopy (EGD). EGD revealed a moderate stenosis in the second portion of the duodenum from compression from the adjacent hematoma. However, the stenosis was able to be traversed into the distal portions of the duodenum.

**Figure 1:**
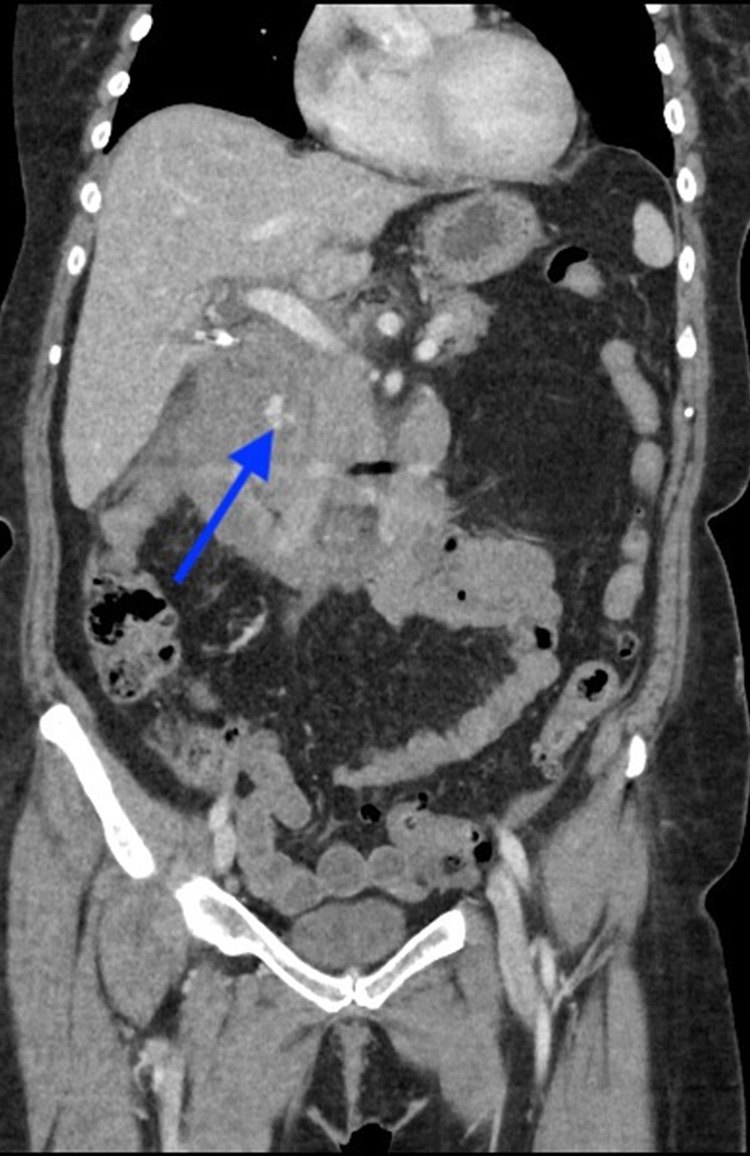
Coronal CT demonstrates a large hematoma in the periduodenal region with a focus of active extravasation (blue arrow).

**Figure 2:**
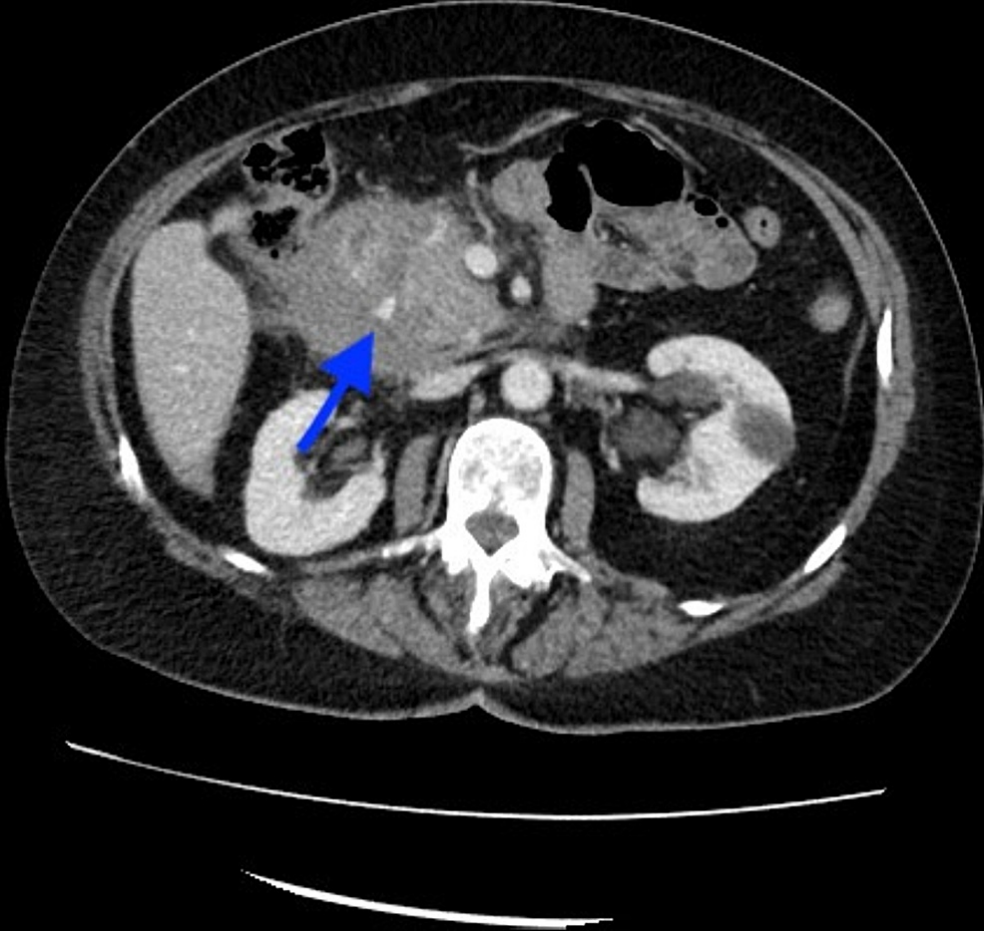
Axial CT demonstrates a large hematoma in the periduodenal region with a focus on active extravasation (blue arrow).

**Figure 3:**
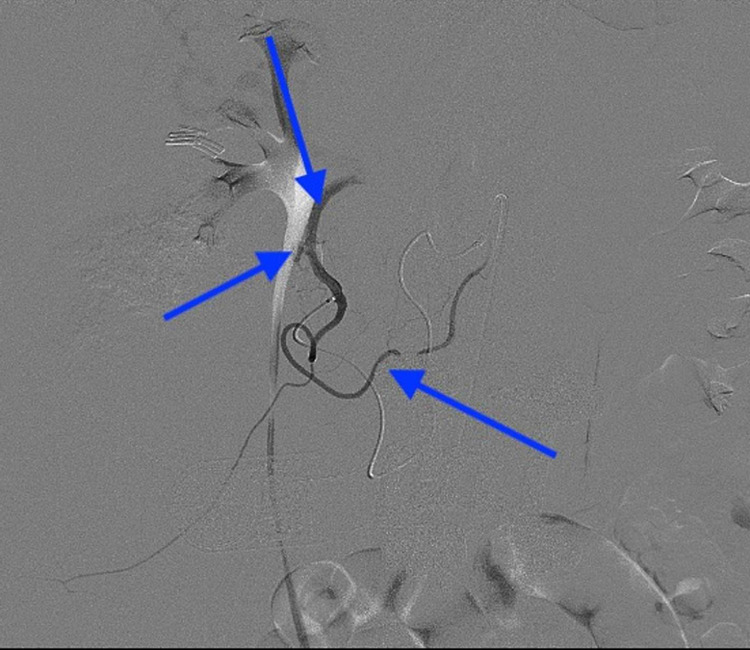
DXA angiography from the superior mesenteric artery with microcatheter selection of the inferior pancreaticoduodenal (bottom arrow) shows active extravasation (middle arrow) from the gastroduodenal artery (top arrow). DXA - diagnostic

The patient remained stable and was able to tolerate a mechanical soft diet prior to discharge. Figures 1-3 show large hematoma in the periduodenal region.

**Figure 4:**
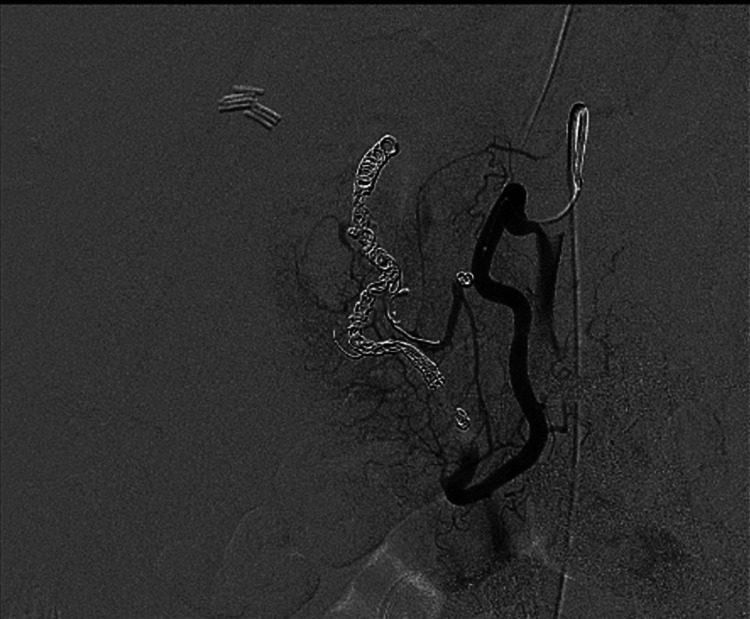
DXA angiography from the superior mesenteric artery with microcatheter selection of the inferior pancreaticoduodenal post coil embolization shows cessation of flow within the embolized branches.

**Figure 5:**
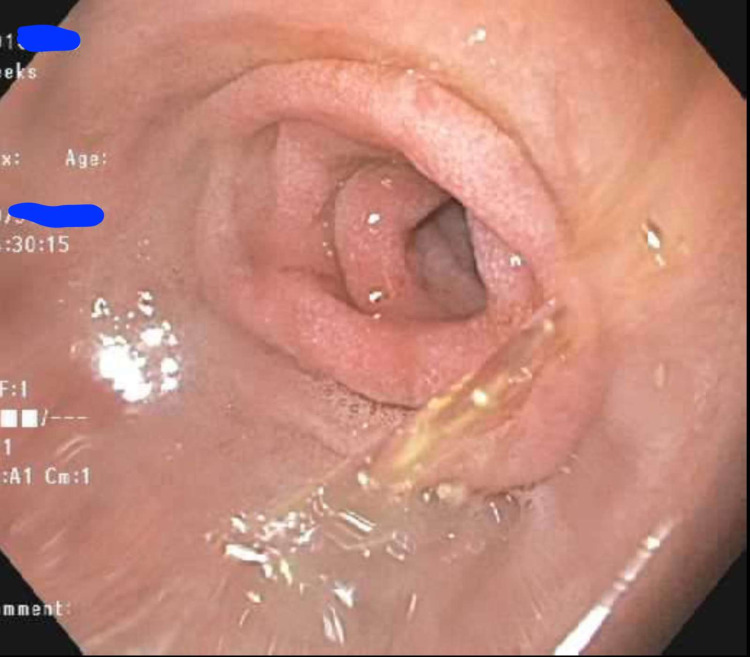
Upper GI endoscopy showing acquired duodenal stenosis second part of duodenum. GI - gastrointestinal

Figure 4 shows post coil embolization, and Figure 5 shows acquired duodenal stenosis of the second part of the duodenum.

 

**Figure 6:**
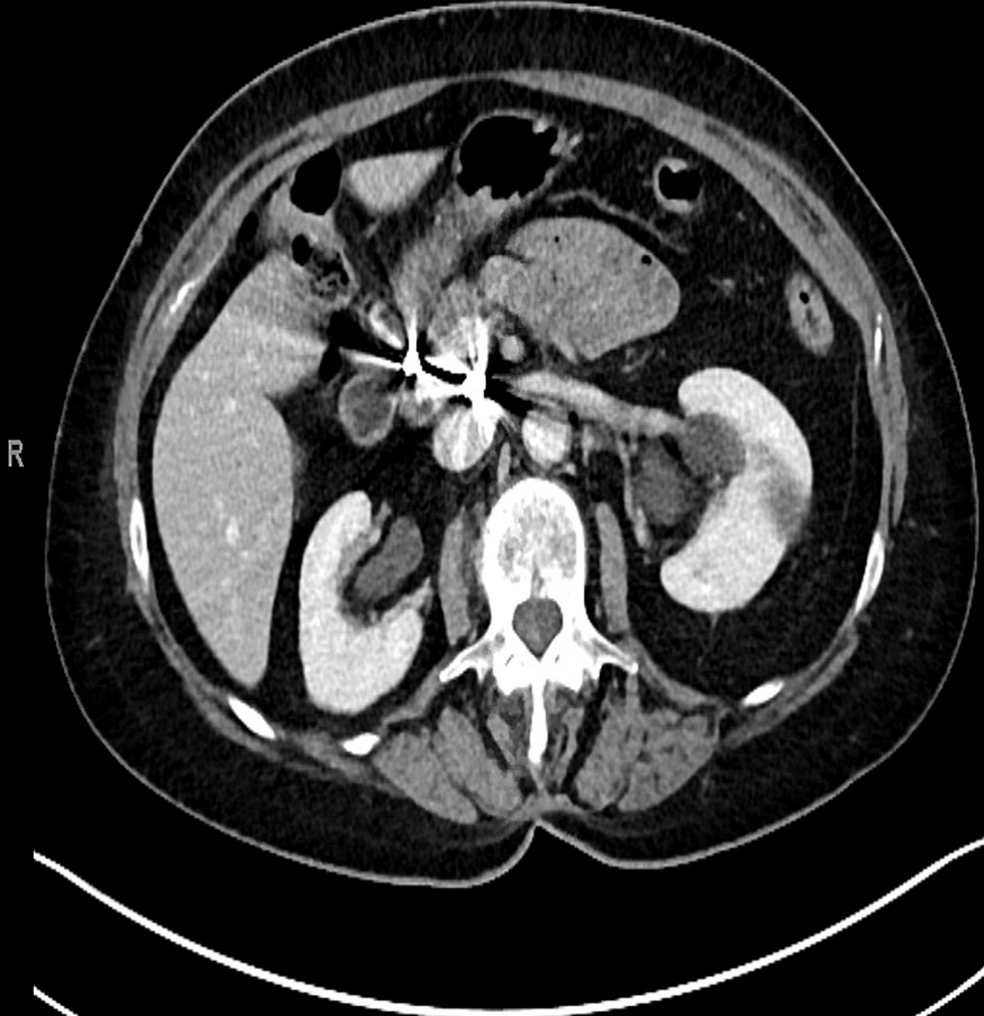
Postprocedural changes of splenic artery aneurysm embolization without evidence of acute complication.

Figure 6 demonstrates postprocedural changes of splenic artery embolization without any acute complications.

## Discussion

The pathogenesis for the development of true aneurysms consists of trauma, hypertension, and atherosclerotic disease [3]. For example, atherosclerosis of the celiac artery results in subsequent stenosis and the development of true GDA aneurysms. More rarely, congenital absence of the celiac axis may also result in aneurysms [4]. The development of collateral vessels as a result of atherosclerosis or congenital absence of blood vessels results in blood vessels being more prone to aneurysmal formation [5].

In a similar fashion, the development of a pancreaticoduodenal artery aneurysm may arise from celiac artery stenosis. Anatomically, the pancreaticoduodenal artery is the main collateral vessel between the celiac axis and the superior mesenteric artery [6]. Therefore, any occlusion of the celiac axis or the superior mesenteric artery may predispose to the formation of a GDA aneurysm [5,7,8].

The most common cause of pseudoaneurysm is related to pancreatitis resulting in vessel wall deterioration [9]. Pseudoaneurysms are mostly a condition of middle age and are mostly found between 50 and 58 years of age [2,10,11]. The most cited locations of pseudoaneurysms include the splenic artery (46%), renal artery (22%), hepatic artery (16.2%), GDA (1.5%), and pancreaticoduodenal artery (1.3%). In a review of literature from 1970 to 1995, the most common cause of GDA aneurysms included pancreatitis (47%), ethanol abuse (25%), peptic ulcer disease (17%), and cholecystectomy (3%) [12-14]. Other potential etiologies included congenital abnormalities such as Marfan syndrome and Ehlers-Danlos syndrome [15], liver cirrhosis [16], and vascular abnormalities such as fibromuscular dysplasia, polyarteritis nodosa, septic emboli, and trauma [5].

Intramural hematoma of the duodenum causing obstruction has been reported with pancreatitis, pancreatic carcinoma, blood dyscrasia, mesenteric cyst, and ruptured aneurysms (Figure 5) [17]. The vast majority of intramural hematomas and intraabdominal hematomas involve blunt trauma (Figures 1-3). Non-penetrating forces, such as blunt trauma, have a greater tendency to affect solid organs or hollow viscera close to their points of fixation. Usually, blunt trauma transmits shearing action impinging the viscus against the spinal column [18]. The duodenum, upper jejunum, and terminal ileum are the most frequent sites of small intestinal injury due to their relative immobility [18].

Intra-abdominal hematoma as a result of trauma usually presents as small bowel obstruction with a delay of symptomatic onset. For the physical exam, a palpable mass is an uncommon finding. As the blood products of hemoglobin undergo protein disintegration, the osmolality of the hematoma increases causing a fluid influx. Over time, the residual intra-abdominal hematoma may cause an increase in extrinsic pressure and result in multiple postoperative complications [18]. This might also help to explain the characteristic delay between injury and the onset of obstructive symptoms.

In terms of clinical signs and symptoms, patients typically present with abdominal pain, nausea, vomiting, and hematochezia. Many of these symptoms overlap with a broad differential diagnosis including small bowel obstruction, gastritis, colitis, or even gastric outlet obstruction. Imaging plays a vital role in narrowing the differential diagnosis. While abdominal x-rays may help to rule out any obstruction, CT scan of the abdomen with contrast provides a more detailed examination of the abdominal etiology.

Spontaneous intramural small-bowel hematomas are usually located within the submucosal layer, and typically cause symptoms after several delays. This delayed onset can be attributed to slow bleeds from small vessels, also resulting in intraluminal, intramesenteric, and retroperitoneal hemorrhages [2,19]. Hemorrhage from submucosal bleeding extending into all layers of the bowel may result in hemorrhagic ascites [2,19].

Literature review

A review of the literature spanning from 1956 to 2011 related 74 incidents of GDA aneurysms, which described hemorrhage as the most common clinical presentation seen in 52% of all cases, followed by abdominal pain seen in 46% of cases [2,20].

Mortality associated with rupture of aneurysms depends upon the anatomical location of the rupture, the severity of the bleed, and the speed of blood loss. Patients with the highest rate of mortality are typically associated with rupture into the duodenum. These patients often present with hematemesis, melena, hemodynamic shock, and have a mortality rate of 21% [2,6,21,22].

Sometimes patients with GDA aneurysm rupture can present with retroperitoneal or intra-peritoneal bleeds with a 19% mortality rate [23-25]. These patients often present with symptoms of gastric outlet obstruction, vomiting, diarrhea, or jaundice secondary to external pressure by the surrounding hematoma [26-28]. Bleeding from the hematoma may also lead to rare instances of recurrent bleeding from the bile ducts or bleeding into the pancreatic duct as hemosuccus pancreaticus [2].

Upon review of literature, one case reported an incident of spontaneous mesenteric hematoma localized to the small bowel of the jejunum. The etiology of the hematoma formation was not identified, and the patient presented with bright red blood per rectum. Emergent laparotomy was pursued rather than interventional mesenteric angiography with embolization given the patients severe anemia and active bleeding [29].

In a case report on imaging findings and outcomes of spontaneous intramural small-bowel hematoma, spontaneous small-bowel hematomas were often single lesions of the jejunum, followed by the ileum and duodenum. Whereas traumatic small bowel hematomas typically affect the duodenum and tend to be more concentrated [2,30]. As supported by radiographic evidence, spontaneous hematomas involve more bowel, usually more than 8 cm in length. The average length of a non-extensive intramural hematoma was 23 cm. Measuring the length may help to distinguish spontaneous intramural small-bowel hematoma from other differentials such as tumor infiltration by metastases or lymphoma [2].

Diagnosis and treatment strategies

After a thorough review of the literature and case reports, a CT scan of the abdomen and pelvis with IV and oral contrast remains the gold standard for imaging and diagnosis of spontaneous intramural-small bowel hematoma. The CT scan may be complemented with barium upper gastrointestinal tract studies with enteroclysis or follow-through of the small bowel. In patients with suspected complete small bowel obstruction, oral contrast material is not needed. Furthermore, water rather than contrast material can provide adequate imaging of the gastrointestinal tract. IV contrast can help to differentiate from other gastrointestinal tract disorders such a malignancy, inflammatory bowel disease, or ischemic and gangrenous gut [2].

Specific MR imaging findings of small bowel hematomas are limited with most of the literature describing duodenal hematomas [2]. In a case that used both computer tomography and magnetic resonance, the hematoma had a well-defined concentric ring configuration. However, MR imaging was able to provide a tissue-specific characterization of the duodenal hematomas [31]. Diagnosis of small bowel obstruction via intra-abdominal hemorrhage may be established with a CT scan and can prevent invasive exploratory abdominal surgery [2].

Treatment options of visceral arterial aneurysms consist of surgical interventions or endovascular interventions consisting of coil embolization and stent placements (Figures 4, 6) [2]. The success rate of open surgeries is 88.2% with a 54-month follow-up [32]. The specific type of intervention depends upon four factors: location of the aneurysm, condition of the patient, presenting symptoms, and the risk of organ ischemia. Emergent surgery remains the treatment of choice in hemodynamically unstable patients consisting of ligation, aneurysmorrhaphy, or bypass surgery [2]. Traditionally, visceral arterial aneurysms were treated with prompt surgical intervention; however, endovascular treatment, such as trans-catheter embolization, has become increasingly popular [33]. However, if the patient experiences a re-bleed or uncontrolled bleeding, vascular surgery may still be a practical option [34].

Vascular reconstruction after the resection of the GDA aneurysm is not always necessary because adequate collateral circulation is usually present from visceral arteries. Unless celiac artery occlusion is present, the GDA and superior mesenteric arteries provide adequate blood supply to the stomach. Ligation of the GDA may cause gangrene of the gallbladder and stomach, and even splenic necrosis [35-37].

Typically, endovascular options of treatment may be performed under local anesthesia, provide a good therapeutic approach for patients with multiple comorbidities. Transcatheter embolization remains the most popular endovascular intervention.

## Conclusions

In summary, intramural hematomas are a relatively rare complication associated with visceral abdominal pseudoaneurysms. Bleeding can be life-threatening requiring emergent intervention. The development of extrinsic compression by the hematoma can manifest in a similar way associated with small bowel obstruction. However, unlike the standard management of small bowel obstruction, the therapeutic approach may require image investigation and prompt endovascular intervention.

## References

[1] Bassaly I, Schwartz IR, Pinchuck A, Lerner R (1973). Aneurysm of the gastroduodenal artery presenting as common duct obstruction with jaundice. Review of literature. Am J Gastroenterol.

[2] Bergert H, Hinterseher I, Kersting S, Leonhardt J, Bloomenthal A, Saeger HD (2005). Management and outcome of hemorrhage due to arterial pseudoaneurysms in pancreatitis. Surgery.

[3] Pitton MB, Dappa E, Jungmann F (2015). Visceral artery aneurysms: Incidence, management, and outcome analysis in a tertiary care center over one decade. Eur Radiol.

[4] Rowsell C, Moore TL, Streutker CJ (2006). Aneurysm of the gastroduodenal artery presenting as a bleeding duodenal ulcer. Clin Gastroenterol Hepatol.

[5] Sachdev U, Baril DT, Ellozy SH (2006). Management of aneurysms involving branches of the celiac and superior mesenteric arteries: a comparison of surgical and endovascular therapy. J Vasc Surg.

[6] Herbert DC (1968). Anticoagulant therapy and the acute abdomen. Br J Surg.

[7] Shanley CJ, Shah NL, Messina LM (1996). Uncommon splanchnic artery aneurysms: pancreaticoduodenal, gastroduodenal, superior mesenteric, inferior mesenteric, and colic. Ann Vasc Surg.

[8] Shikata D, Nakagomi H, Takano A (2016). Report of a case with a spontaneous mesenteric hematoma that ruptured into the small intestine. Int J Surg Case Rep.

[9] Schweizer W, Gries NC, Maddern G, Triller J (1999). Splenic infarction complicating ligation of a gastroduodenal artery aneurysm. Dig Surg.

[10] Coll DP, Ierardi R, Kerstein MD, Yost S, Wilson A, Matsumoto T (1998). Aneurysms of the pancreaticoduodenal arteries: a change in management. Ann Vasc Surg.

[11] Dinter DJ, Rexin M, Kaehler G, Neff W (2007). Fatal coil migration into the stomach 10 years after endovascular celiac aneurysm repair. J Vasc Interv Radiol.

[12] Eckhauser FE, Stanley JC, Zelenock GB, Borlaza GS, Freier DT, Lindenauer SM (1980). Gastroduodenal and pancreaticoduodenal artery aneurysms: a complication of pancreatitis causing spontaneous gastrointestinal hemorrhage. Surgery.

[13] Ferrero E, Ferri M, Viazzo A (2011). Visceral artery aneurysms, an experience on 32 cases in a single center: treatment from surgery to multilayer stent. Ann Vasc Surg.

[14] Germanos S, Soonawalla Z, Stratopoulos C, Friend PJ (2009). Pseudoaneurysm of the gastroduodenal artery in chronic pancreatitis. J Am Coll Surg.

[15] Gouny P, Fukui S, Aymard A, Decaix B, Mory H, Merland JJ, Nussaume O (1994). Aneurysm of the gastroduodenal artery associated with stenosis of the superior mesenteric artery. Ann Vasc Surg.

[16] Grotemeyer D, Duran M, Park EJ (2009). Visceral artery aneurysms--follow-up of 23 patients with 31 aneurysms after surgical or interventional therapy. Langenbecks Arch Surg.

[17] Habib N, Hassan S, Abdou R (2013). Gastroduodenal artery aneurysm, diagnosis, clinical presentation and management: a concise review. Ann Surg Innov Res.

[18] Bohl JL, Dossett LA, Grau AM (2007). Gastroduodenal artery pseudoaneurysm associated with hemosuccus pancreaticus and obstructive jaundice. J Gastrointest Surg.

[19] Moore E, Matthews MR, Minion DJ, Quick R, Schwarcz TH, Loh FK, Endean ED (2004). Surgical management of peripancreatic arterial aneurysms. J Vasc Surg.

[20] Harris K, Chalhoub M, Koirala A (2010). Gastroduodenal artery aneurysm rupture in hospitalized patients: an overlooked diagnosis. World J Gastrointest Surg.

[21] Iyori K, Horigome M, Yumoto S (2004). Aneurysm of the gastroduodenal artery associated with absence of the celiac axis: report of a case. Surg Today.

[22] Iyomasa S, Matsuzaki Y, Hiei K, Sakaguchi H, Matsunaga H, Yamaguchi Y (1995). Pancreaticoduodenal artery aneurysm: a case report and review of the literature. J Vasc Surg.

[23] Kueper MA, Ludescher B, Koenigsrainer I, Kirschniak A, Mueller K, Wiskirchen J, Koenigsrainer A (2007). Successful coil embolization of a ruptured gastroduodenal artery aneurysm. Vasc Endovascular Surg.

[24] Lee PC, Rhee RY, Gordon RY, Fung JJ, Webster MW (1999). Management of splenic artery aneurysms: the significance of portal and essential hypertension. J Am Coll Surg.

[25] Matsuno Y, Mori Y, Umeda Y, Imaizumi M, Takiya H (2008). Surgical repair of true gastroduodenal artery aneurysm: a case report. Vasc Endovascular Surg.

[26] Jones WR, Hardin WJ, Davis JT, Hardy JD (1971). Intramural hematoma of the duodenum: a review of the literature and case report. Ann Surg.

[27] Kasirajan K, Greenberg RK, Clair D, Ouriel K (2001). Endovascular management of visceral artery aneurysm. J Endovasc Ther Offic J Inter Soc Endovasc Specialists 2001,8(2): 150-155. Epub.

[28] Koyazounda A, Jaillot P, Persico J, Thouret JM, Grand A (1994). Aneurysm of the gastroduodenal artery ruptured into the peritoneum. Treatment by embolization. (Article in French). Presse Med.

[29] Hahn PF, Stark DD, Vici LG, Ferrucci JT Jr (1986). Duodenal hematoma: the ring sign in MR imaging. Radiology.

[30] Matsuzaki Y, Inoue T, Kuwajima K (1998). Aneurysm of the gastroduodenal artery. Intern Med.

[31] Morita Y, Kawamura N, Saito H, Shinohara M, Irie G, Okushiba S (1988). Diagnosis and embolotherapy of aneurysm of the gastroduodenal artery. (Article in Japanese). Rinsho Hoshasen Clin Radiography.

[32] Sessa C, Vokrri L, Porcu P, Maurin M, Stahl JP, Magne JL (2005). Abdominal aortic aneurysm and Coxiella burnetii infection: report of three cases and review of the literature. J Vasc Surg.

[33] Sessa C, Tinelli G, Porcu P, Aubert A, Thony F, Magne JL (2004). Treatment of visceral artery aneurysms: description of a retrospective series of 42 aneurysms in 34 patients. Ann Vasc Surg.

[34] Takahashi T, Shimada K, Kobayashi N, Kakita A (2001). Migration of steel-wire coils into the stomach after transcatheter arterial embolization for a bleeding splenic artery pseudoaneurysm: report of a case. Surg Today.

[35] Tulsyan N, Kashyap VS, Greenberg RK, Sarac TP, Clair DG, Pierce G, Ouriel K (2007). The endovascular management of visceral artery aneurysms and pseudoaneurysms. J Vasc Surg.

[36] Vogler C, Faiss J, Krause FJ, Schwamborn G (1991). [Aneurysm of the gastroduodenal artery with aplasia of the celiac trunk]. Der Chirurg Zeitschrift fur alle Gebiete der operativen Medizen.

[37] White AF, Baum S, Buranasiri S (1976). Aneurysms secondary to pancreatitis. AJR Am J Roentgenol.

